# Cannulation for Neonatal and Pediatric Extracorporeal Membrane Oxygenation for Cardiac Support

**DOI:** 10.3389/fped.2018.00017

**Published:** 2018-03-19

**Authors:** Chris Harvey

**Affiliations:** ^1^University Hospitals of Leicester, Leicester, United Kingdom

**Keywords:** extracorporeal membrane oxygenation, cannulation, veno-arterial, veno-venous, pediatric cardiac support, neonatal cardiac support

## Abstract

The use of extracorporeal membrane oxygenation (ECMO) has increased over recent years providing respiratory and cardiac support. Optimal cannula placement is essential for successful patient outcomes. Multiple cannulation strategies may be employed depending on the age/weight of the patient and their underlying condition. This article discusses cannulation technique focusing on the cannulation of pediatric and neonatal patients for cardiac support on ECMO.

## Introduction

Extracorporeal membrane oxygenation (ECMO) is a method of supporting the heart and or the lungs while native organ function recovers. The use of ECMO has increased significantly over recent years (1). 6,960 neonatal and pediatric cardiac ECMO runs have been reported to ELSO as of 2016 and a further 1,779 cases of ECPR in the same age group. Survival to discharge in this group for cardiac neonates is 42% and pediatric 41% (1). While survival from cardiac ECMO is not as good as respiratory disease [neonates 74% and pediatric 58%, ELSO Registry (1)], it has to be remembered that without cardiac ECMO the mortality for many of these patients would approach 100%. With improvement in both oxygenators and pump technology the management of patients on ECMO has become simpler but one prerequisite to a successful ECMO run that remains is the accurate and safe placement of suitably sized cannula. The exact cannulation method used depends on the desired mode of ECMO, the size and age of the patient, and the experience of the cannulating physician. The following article will focus on cannulation of both pediatric and neonatal patients for cardiac assist as this is a group of patients in whom a single approach to cannulation is not always applicable and most inter patient variation exists.

## Pre-Cannulation Considerations

### Cannula Design

Access to a variety of cannula of differing size is essential when treating children. A guide to the required cannula size is shown in Table 1. The design of the cannula should maximize blood flow for a given diameter without compromising cannula strength or function from too thin or weak a cannula wall. Wire reinforcement is useful and can reduce the risk of cannula collapse and or kinking (2). While it is important to acknowledge Poiseuille’s law that states that flow of a fluid through a tube is proportional to the fourth power of the radius times the pressure divided by the length it has to be remembered that there is often little to be gained from simply placing the largest cannula possible into a vessel. A large cannula is more prone to damage the vessel or lead to venous obstruction and unless the cannula enters a large reservoir of blood such as the right atrium the side drainage holes in the cannula may be obstructed by the close proximity to the venous wall. Obstruction to side holes can lead to intermittent drainage with associated swings in pressure and increase the risk of hemolytic complications.

**Table 1:** Cannula size (Fr) by patient weight.

Patient size (kg)	Arterial cannula (Fr)	Venous cannula (Fr)
2	8	8–10
3–6	10	10–12
6–8	12	14
8–16	14	17
16–30	17	19
30–40	17	21
>40	21	25

In addition, if ECMO is to be delivered utilizing a centrifugal pump, then the achieved flow is dependent on both the inlet flow (pre-load) and the post pump pressure (afterload). Therefore, there is no benefit in placing a large venous cannula if the arterial cannula is small. For example, if placing a 10-Fr arterial cannula then either a 10- or a 12-Fr venous cannula will provide adequate drainage. Placement of a 14-Fr venous cannula may predispose to unsuccessful placement and resultant damage to the vessel.

## Mode of Support

### Veno-Arterial (VA) Cannulation

#### Technique—Neonates

**Figure 1:**
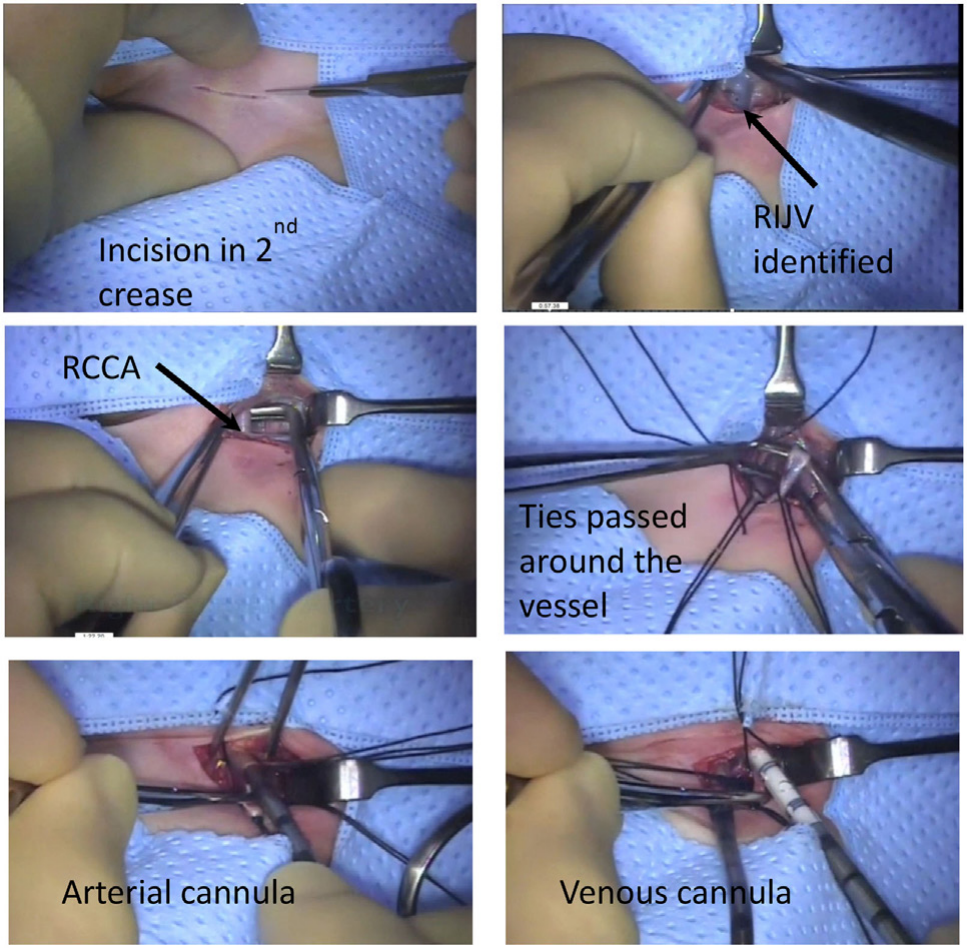
Neonatal veno-arterial cannulation steps.

Most neonates should be cannulated from the right neck. This allows both VA and veno-venous cannulation. The baby should be positioned on a small roll placed under the neck. The neck should be extended and tilted approximately 30–45° to the left. The skin is prepped and drapes applied. A clear plastic drape allows the ET tube to be visualized and can aid in the reduction of patient instability due to a kinked ET tube. The skin is incised along the second skin crease about 1.5–2 cm above the clavicle. The incision need only be 1–2 cm in length and is centered on the medial border of sternocleidomastoid muscle (SCM) (Figure 1). The right internal jugular vein and common carotid artery are identified by retracting the SCM laterally. The anterior belly of omohyoid is encountered deep to the SCM and may be divided if required. Care must be taken to avoid damaging the vagus nerve that runs between the two vessels. It is important that on first visualizing the vessels an assessment of their caliber and, therefore, the expected cannula size should be made. With further dissection and handling the vein especially is prone to collapse making accurate sizing at a later stage inaccurate. Heavy braided ties (2-0 or No 1) are passed around the vessels both above and below the intended cannulation site, these will be used to prevent bleeding at the cannulation site and to secure the cannula post insertion. A bolus of anticoagulant should be given at this stage and allowed to circulate fully. Unfractionated heparin is the commonest anticoagulant and should be given at a dose of between 50 and 100 IU per kg (3). The artery is tied cranially and a vascular clamp applied caudally. The vessel is opened with a transverse arrow head incision; care being taken only to open the anterior half of the vessel. The cannula is introduced and the vascular clamp removed. A gentle rocking or twisting of the cannula along its long access can aid entry into the artery and reduce the occurrence of local trauma. The cannula should be inserted 2–3 cm and firmly secured in place with the braided ties. Some centers advocate placing a small piece of silastic between the suture and the vessel to make it easier to cut the tie at decannulation but this is not essential. Once secure, the cannula should be connected to the ECMO circuit ensuring no air bubbles are trapped in the tubing. Flushing the cannula will reduce the risk of developing clot in the cannula until ECMO is established.

The process is repeated for the venous cannula. Again the vessel is ligated with the braided ties cranially and vascular clamp applied caudally. As the vein is particularly prone to collapse, great care must be taken to ensure that the venotomy only involves the anterior half of the vessel. A deeper incision may lead to complete transection of the vessel during insertion of the cannula. If this occurs then small 7-0 prolene stay sutures may facilitate control and opening of the lumen of the vein. For most neonates, insertion of the cannula to a depth of 6–7 cm is usually adequate. The venous cannula is connected to the circuit again insuring that this is air free and ECMO can then be commenced. The wound is closed and further securing sutures inserted externally avoiding excessive tension on the skin and the resultant risk of necrosis.

It is possible to cannulate the left side of the neck but this is rarely required and if the right side is not utilized due to vessel occlusion may lead to increased cerebral complication either through increased venous pressure or decreased arterial flow. Cannulation of the femoral vessels should be avoided as the vessel is often too small below 10 kg and both the limb and the ECMO support could be compromised.

While not a common site of cannulation for central access in neonates the presence of a pre-existing right internal jugular vein, central line should not preclude the use of this vessel for ECMO. Alternative central access should be sought especially if the child is unstable and requiring significant inotropic support prior to commencing cannulation for ECMO. The vessel is dissected in the same manner and if the entry of the central line is evident then this should be used as the site for ECMO cannulation. If the central line enters the vein outside the cannulation field, it is safe to remove the line. Placing a single suture through the skin at the entry point of the central line is sufficient to provide hemostasis and removes the need to press on the vessel. Cannulation may then proceed as described above.

### Difficult Cannulation

Some cannulas have the option of utilizing a guidewire and introducer as opposed to a blind ending stylet. The insertion of a guidewire and introducer may help with cannula placement after an initial failed attempt. Echo and/or fluoroscopy can be useful to confirm the position of the wire in the appropriate major vessel or the atria prior to inserting the cannula and the resulting cannula position (4). The use of a guidewire may be useful if a false lumen has been created after a failed attempt or in those patients in whom the cannula can be advanced only a few centimeters before catching on either the subclavian or azygous vein.

An alternative approach if the right internal jugular vein is damaged at initial insertion of the venous cannula is to surgically dissect the vessel caudally. By following the right internal jugular vein toward the chest fresh vein can be uncovered and a further attempt at cannula insertion is possible. If required, the cannula can be inserted at the junction of the subclavian and jugular veins. However, care must be taken not to disrupt venous drainage to the arm both during ECMO, by obstruction with the cannula. Following decannulation, the vein requires careful repair as to prevent stenosis to the subclavian vein and post ECMO venous obstruction.

### Special Considerations

Most patients requiring ECMO will have had an echocardiogram as part of their work up; however, if the vein is found to be significantly smaller than the artery on initial inspection, then a left SVC may be present and repeat echocardiogram may be beneficial.

**Figure 2:**
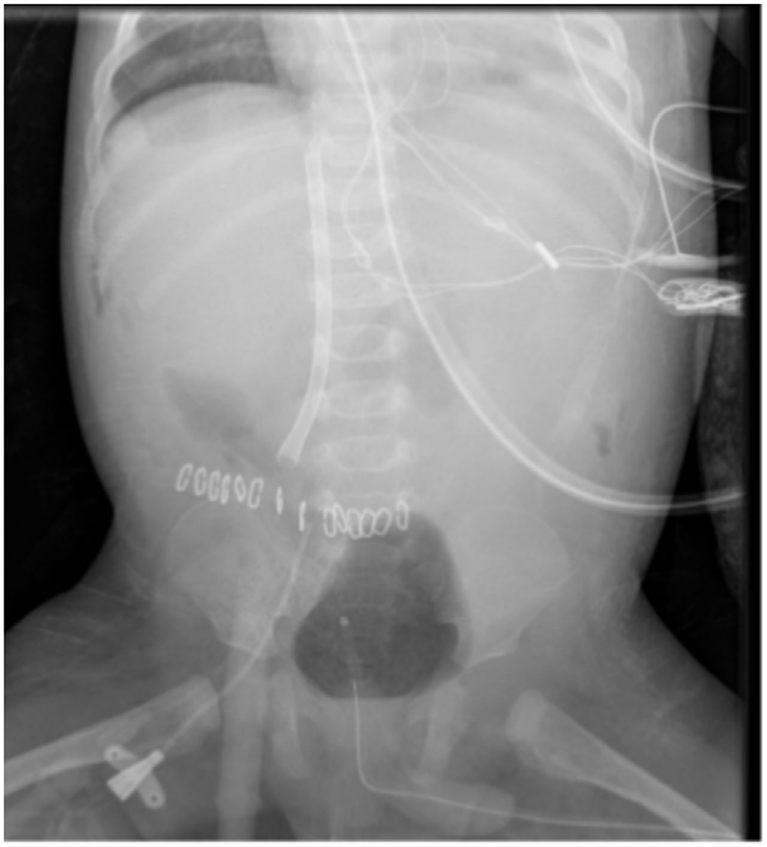
IVC access *via* extra-peritoneal approach.

Cannulation following bidirectional Glenn may also be difficult due to the separation of the venous blood returning from the upper and lower body. It was thought that the outcome in these patients was poor but more recent data from the ELSO registry suggest support in these patients is a viable option (5). If ECMO is required in the peri-operative period then central cannulation may be easiest with direct access to the right atrium. However, if peripheral cannulation is desired and the patient is too small to drain the lower body from the femoral vessels then the abdominal IVC may be accessed directly. An incision is made over the anterior abdominal wall midway between the umbilicus and the anterior superior iliac spine. Dissection is then continued posteriorly in the extra-peritoneal plane until the IVC is identified. A small purse string is inserted into the vein and a cannula can is then inserted. The cannula should reach the right atrium from below (Figure 2). A cannula can then be placed from above into the Glenn to decompress the superior vena cava and reduce cerebral venous congestion. Other potential methods of cannulation include the use of a dual-lumen catheter placed directly through the right atrial wall (6) and percutaneous trans-hepatic cannulation of the IVC (7).

Patients with systemic-pulmonary shunts also need consideration when cannulating for ECMO. During ECMO, traditionally the shunt was restricted to reduce pulmonary run off and to increase systemic perfusion. Prior to trialing off ECMO, the shunt would then need to be unclipped and reclipped if the trial proved unsuccessful. However, by maintaining higher than normal ECMO flows (150–200 ml/kg) systemic perfusion can be maintained without the need to restrict the shunt. This approach has been shown to yield similar outcomes to non-shunt patients (8).

### Central VA Cannulation

Cannulation centrally through the chest is a viable option and is commonly used following failure to wean from bypass. Some have advocated the use of high flow central ECMO in patients in septic shock (9). Central ECMO allows larger diameter cannula to be placed with the potential for increased flow. If ECMO is required following failure to wean from bypass, then it is often easier to connect the circuit to the pre-existing cannula. Time needs to be spent ensuring that the cannulas remain secure and the applied purse strings are hemostatic. If more prolonged ECMO support is envisaged, then the patient should be cannulated for peripheral ECMO as this allows more stable support, less likelihood of bleeding (10), and the ability to formally close the chest.

### Veno-Venous Cannulation

While veno-venous (VV) ECMO is most commonly reserved to treat primary respiratory failure, it can be used to provide support for patients with complex congenital heart conditions as well (11). It is especially useful in those patients with a univentricular anatomy or other shunt-dependent circulations in whom the problem may not be one of primary cardiac pump failure but simply of insufficient pulmonary blood flow with resultant hypoxia. Cannulation for VV ECMO avoids damage to the carotid artery while providing oxygen into the circulation. In addition, VV ECMO does not cause alterations in cardiac filling or increased afterload that is seen with VA support and prevents the need for cardiac venting.

Cannulation for VV ECMO may be achieved through a full percutaneous approach and is best performed with the aid of ultrasound both to size and then cannulate the vessel. It is our practice to cannulate for VV ECMO by using a modified Seldinger technique (12). The neck is incised as with VA cannulation in the second cervical skin crease and the jugular vein is identified. Only the anterior portion of the vein is cleared of supporting fascia. The vein is cannulated from above through a separate incision with a 20-G cannula. A soft guidewire is then inserted and a standard vascular access sheath (4–6 Fr) is inserted into the vein. A larger diameter guidewire is then inserted into the right atrium through the sheath. The use of fluoroscopy and or echocardiography can be useful to confirm the presence of the wire in the heart or IVC. If the plan is to utilize a bicaval cannula, then the wire needs to be placed into the IVC with the tip left just below the level of the renal veins. Tract is then dilated and heparin bolus given. The use of a small artery clip to further open the skin incision may facilitate passage of the cannula. The cannula is inserted to the desired depth and ECMO commenced. The cannula is secured with sutures and the wound closed.

### Complications of VV Cannualtion

In our institution, we have moved away from using the bicaval double-lumen cannula due to late perforations (13) in favor of peripheral VA cannulation. 5 out of the 78 neonates cannulated with a 13-Fr bicaval cannula suffered a cardiac perforation and subsequent cardiac arrest from tamponade. The perforations were seen following a normal echocardiogram immediately post cannulation and ranged from between 3 and 10 days post insertion. All patients survived the arrest.

### Pediatric Patients

For patients above the age of 2 years and >10 kg in weight, then VA support is most commonly achieved through the femoral vessels. Both surgical and percutaneous cannulation techniques may be utilized successfully.

### Surgical Cannulation of the Femoral Vessels

The patient is prepped and draped. An incision is made centered over the femoral artery. Either a transverse incision or a longitudinal incision can be utilized dependent on the preferences of the surgeon. The common femoral artery is exposed and both the profunda and superficial femoral arteries identified and dissected. A bolus of heparin is given. The artery may be cannulated directly but our preferred method is to insert the cannula utilizing the modified Seldinger technique. The use of the Seldinger approach may permit sufficient distal flow to negate the need for an additional cannula as it avoids the need for a purse string and narrowing of the vessel. The common femoral artery is entered below the level of the inguinal ligament with the needle being passed through a separate stab incision placed on the upper thigh this is the wired for the cannula. It is possible to puncture the artery directly and then pass the guidewire in a retrograde fashion through the leg. The vein can be cannulated on the ipsilateral side again through a separate stab incision. The cannulas are secured and ECMO commenced.

### Distal Limb Perfusion

**Figure 3:**
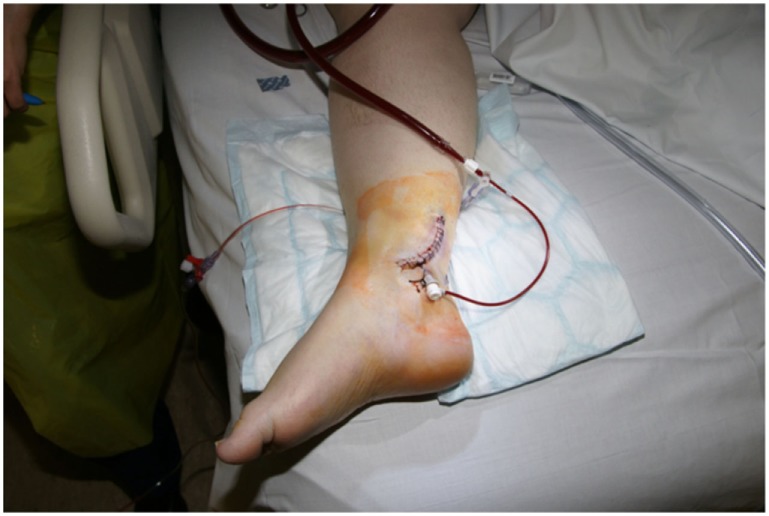
Cannulation of posterior tibial artery allowing retrograde perfusion of limb.

Immediately after cannulation, thought must be given to distal limb perfusion. Limb ischemia is a major problem of VA ECMO support (14). This may be achieved through various methods. The limb should be assessed clinically with the aid of saturation monitoring (pulse oximetry probe placed on the limb distal to the site of cannulation) and near-infrared spectroscopy (15). If perfusion is felt adequate, then observation of the limb may suffice. The second approach is to place a cannula in the superficial femoral artery, this may be achieved at open dissection or percutaneously even in small children (16). This cannula is then connected to the ECMO circuit through a luer lock connector. A vascular access sheath may provide sufficient flow but more reliable flow may be achieved by using a neonatal arterial cannula of between 8 and 10 Fr. Third, a cannula may be placed in a distal artery, usually the posterior tibial artery, with the limb being perfused in a retrograde manner (Figure 3). This can be achieved with an ultrasound guided percutaneous technique but is often best achieved through a cut down.

### Vessel Sparing Cannulation

While not appropriate in the acute situation especially when the patient is in cardiac arrest, flow to the distal limb can be maintained by the anastomosis of a graft to the femoral artery. Again the artery is exposed and an 8–10 mm graft is sewn on to the vessel at 45°. The ECMO tubing can then be tunneled through the skin and the circuit can then be attached directly to the graft *via* a connector (a 1/4″ connector fitting the 8 mm graft and a 3/8″ the 10 mm). Bidirectional flow is then achieved into the limb. Occasionally, flow distally into the limb may be too high causing edema and swelling of the limb. If this occurs, a constricting band can be placed distally to the anastomosis restricting inflow into the leg. This technique can be applied to cannulation of the upper limb arteries, both the axillary and subclavian arteries have been used for this method although a higher incidence of hyperperfusion has been reported in the these vessels (17).

## Venting

**Figure 4:**
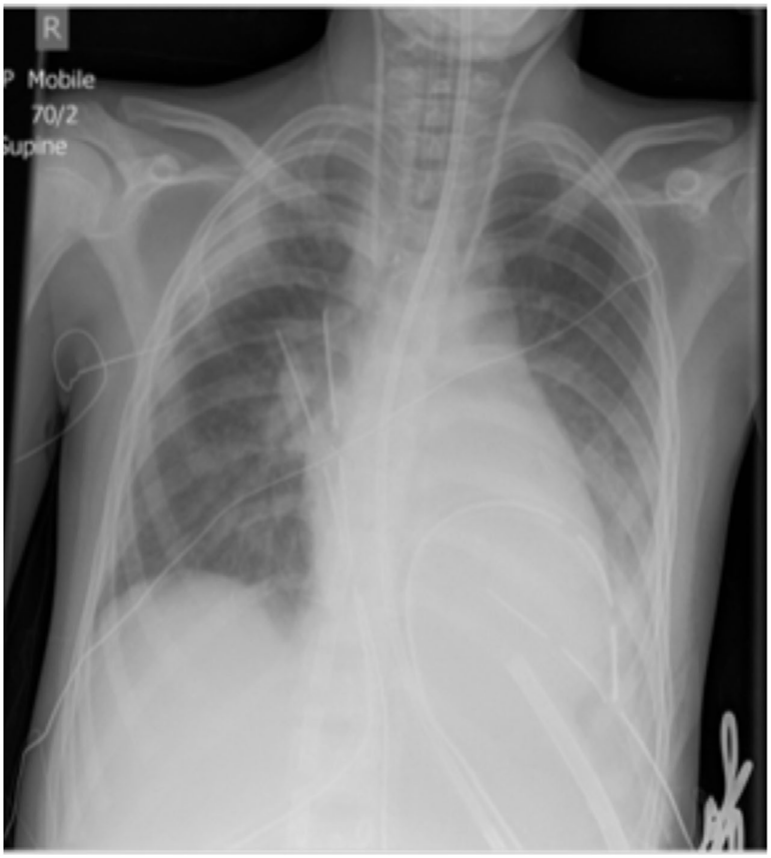
LV Vent.

After cannulation for VA ECMO in children, consideration should be given to venting the left heart to prevent distention, subendocardial ischemia, and pulmonary edema. The options available depend on the age and size of the patient, whether the chest is open or closed and the anticipated length of the ECMO run. Options include septostomy, insertion of a superior pulmonary venous cannula, insertion of a peripheral cannula placed trans-septally into the left atrium (LA) or placement of a vent through the LV apex (Figure 4).

### Chest Open Venting

If the chest is opened, then a cannula can be placed into the LA *via* the superior pulmonary vein. The cannula can be tunneled to permit temporary closure of the chest. Flow may be limited through the vent and in neonates they are prone to clotting off especially as cardiac function improves and LA pressures drop. It may be necessary to partially occlude the main venous drainage ECMO cannula to achieve sufficient flow down the vent to maintain patency. Removal of the vent is surgical and requires the chest to be open.

Occasionally, despite the presence of a LA vent the ventricle may remain distended it may be necessary to place a larger cannula directly into the left ventricle. This can be achieved *via* a left thoracotomy and the chest closed.

### Chest Closed Venting

If the chest is closed then the option of a peripherally inserted cannula into the LA or septostomy are preferred. If a patent foramen ovale exists, it is relatively simple for the cardiologist to enlarge this with a balloon. This can be performed at the bed side and may provide sufficient decompression under echo control. If the septum is intact, then the patient is transferred to the cardiac catheter suite for a trans-septal perforation and stent insertion (18). Direct needle perforation and radiofrequency have been used to perforate the septum and it is our practice to follow this with a stent to maintain patency of the new shunt. The second option for a closed chest is to place a drainage cannula across the septum from the groin. Cannulas of up to 22 Fr have been safely placed in adults in this manner (19).

## Conclusion

Extracorporeal membrane oxygenation (ECMO) is a valuable tool to provide short-term cardiac support either as a means to allow recovery. Various techniques exist to cannulate neonates and children and by utilizing the correct approach most patients can be supported safely.

## Author Contributions

The author confirms being the sole contributor of this work and approved it for publication.

## Conflict of Interest Statement

The author declares that the research was conducted in the absence of any commercial or financial relationships that could be construed as a potential conflict of interest.
